# Derivation of Soil Ecological Criteria for Copper in Chinese Soils

**DOI:** 10.1371/journal.pone.0133941

**Published:** 2015-07-24

**Authors:** Xiaoqing Wang, Dongpu Wei, Yibing Ma, Mike J. McLaughlin

**Affiliations:** 1 Department of Environmental Engineering and Chemistry, Luoyang Institute of Science and Technology, Luoyang, P. R. China; 2 Institute of Agricultural Resources and Regional Planning, Chinese Academy of Agricultural Sciences, Beijing, P. R. China; 3 School of Resources and Environment, University of Jinan, Jinan, China; 4 Centre for Environmental Contaminant Research, CSIRO Land and Water, Adelaide, Australia; 5 Soil Science, Waite Research Institute, School of Agriculture Food and Wine, University of Adelaide, Adelaide, Australia; Chinese Research Academy of Environmental Sciences, CHINA

## Abstract

Considerable information on copper (Cu) ecotoxicity as affected by biological species and abiotic properties of soils has been collected from the last decade in the present study. The information on bioavailability/ecotoxicity, species sensitivity and differences in laboratory and field ecotoxicity of Cu in different soils was collated and integrated to derive soil ecological criteria for Cu in Chinese soils, which were expressed as predicted no effect concentrations (PNEC). First, all ecotoxicity data of Cu from bioassays based on Chinese soils were collected and screened with given criteria to compile a database. Second, the compiled data were corrected with leaching and aging factors to minimize the differences between laboratory and field conditions. Before Cu ecotoxicity data were entered into a species sensitivity distribution (SSD), they were normalized with Cu ecotoxicity predictive models to modify the effects of soil properties on Cu ecotoxicity. The PNEC value was set equal to the hazardous concentration for x% of the species (HCx), which could be calculated from the SSD curves, without an additional assessment factor. Finally, predictive models for HCx based on soil properties were developed. The soil properties had a significant effect on the magnitude of HCx, with HC5 varying from 13.1 mg/kg in acidic soils to 51.9 mg/kg in alkaline non-calcareous soils. The two-factor predictive models based on soil pH and cation exchange capacity could predict HCx with determination coefficients (R^2^) of 0.82–0.91. The three-factor predictive models – that took into account the effect of soil organic carbon – were more accurate than two-factor models, with R^2^ of 0.85–0.99. The predictive models obtained here could be used to calculate soil-specific criteria. All results obtained here could provide a scientific basis for revision of current Chinese soil environmental quality standards, and the approach adopted in this study could be used as a pragmatic framework for developing soil ecological criteria for other trace elements in soils.

## Introduction

Copper (Cu) is an essential nutrient for plant growth [1,2]; however, it may become phytotoxic and cause metabolic disorders at high soil concentration [3]. As a well-known active ingredient of fungicide and animal feeds, Cu has been widely used in agriculture and livestock breeding in recent decades, and this has resulted in elevated Cu concentrations in agricultural soils. Many countries and jurisdictions have developed soil environmental quality standards (EQSs) or their equivalent to protect human health and terrestrial ecosystems from trace metals (including Cu) that were deliberately or inadvertently added to soil. Cu has specific properties that are important with regard to setting EQS. First, excessive Cu in soils led to toxicity of plant roots and less transportation from roots to leaves and grains, so that Cu toxicity occurs often in ecology and less in plant food safety because the Cu concentration in the edible part of plant does not often exceed the acceptable level [4]. EQS for Cu is intended to be ecologically meaningful and defined on the basis of sound scientific information, its concentrations in the environment with no negative impact on terrestrial ecosystems and ecotoxicological principles. Second, Cu is an essential element that naturally occurs in the environment, concentrations of Cu in soil consists of both natural pedo-geochemical and anthropogenic fractions [5]. The background concentration of Cu in soil may be beneficial or essential to terrestrial ecosystems. Use of a single EQS limit value, regardless of the Cu lability difference between background and anthropogenic fractions, could result in either overestimation or underestimation of metal contamination and the associated risk for a particular soil. The added risk approach, described in detail by Struijs et al. [6], was proposed to improve the derivation of soil ecological criteria for metals used to set EQS. The added risk approach is based on the idea that the background concentration of a naturally occurring substance should be accepted as posing no risk to the environment. The Ministry of Housing, Spatial Planning and the Environment (Netherlands) has applied the criteria derived by the added risk approach for Cu, cadmium, lead and zinc in water and soil to set EQSs [7].

Available ecotoxicity data most come from single-species ecotoxicity tests measuring effects on individual species. However, populations, communities and ecosystems are generally the entities to be protected and the sensitivities to particular contaminant’s ecotoxicity differ among species. To resolve this incongruity between individual-based data and the complex biological entities, the species sensitivity distribution (SSD) method was proposed [8]. Various species sensitivities towards a chemicals can be captured in a variability distribution, called the SSD. From a SSD, a hazardous concentration is identified at which a certain percentage (x) of all species is assumed to be affected [percentage (100 –x) to be protected]. The SSD represents the variation in sensitivity of species towards a contaminant by a statistical or empirical distribution function of responses for a set of species [9]. With its greater statistical significance and ecological meaning, SSD is increasingly applied in soil ecological criteria setting and risk assessment [10–12]. Recently, a research series concerning Cu ecotoxicity for native terrestrial species based on Chinese soils was performed [13–16], which can not only accurately predict the ecotoxicity of Cu as a function of soil properties but also enriched the Cu chronic ecotoxicity data for deriving soil ecological criteria. Although it is well known that soil properties play an important role in modifying ecotoxicity of metals [17–23], and the quantitative relationships between metals’ ecotoxicity and soil properties have been developed [24–26], few regulations for protecting the soil environment have adopted such scientific information. Additionally, the discrepancy between ecotoxicological datasets for different laboratory and field conditions should be corrected or normalized [23]. Therefore, the approach that integrates bioavailability/ecotoxicity, species sensitivity and differences in laboratory and field ecotoxicological datasets, was used in the present study to derive the ecological criteria for Cu as a function of soil properties and to provide a scientific basis for revision of the Chinese current soil EQSs. The adopted approach could be used as a pragmatic framework for developing ecological criteria for other trace elements in soils.

## Materials and Methods

**Fig 1:**
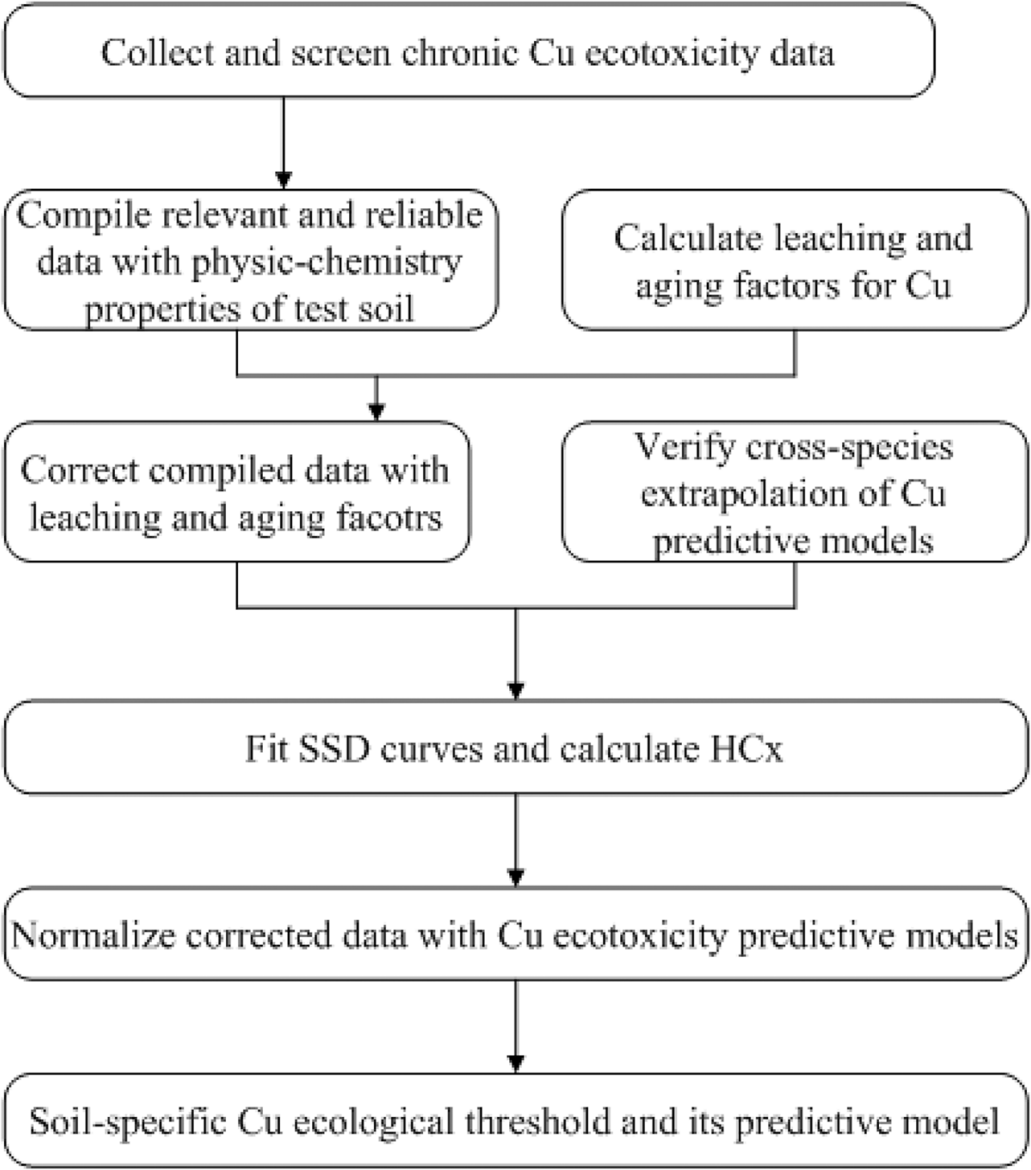
Schematic framework used for derivation of soil Cu ecological criteria.

A schematic overview for Cu ecological criteria derivation is given in Fig 1.

### Ecotoxicological data collection and compilation

The ecotoxicological data (the concentrations that cause 10% inhibition effect, EC10) for Cu were retrieved or calculated from the raw data reported in all available peer-reviewed literature (Tables A and B in S1 File), in which ecotoxicity tests were carried out on Chinese soils. First, the obtained data were screened for reliability and relevancy. Reliability mainly covered the methodology and the way that the performance and results of the test were described. Relevancy related to the soil used in the ecotoxicity test and the possibility of obtaining or estimating the soil properties. Second, the Cu ecotoxicity predictive models were used to describe the relationship between ecotoxicity and soil properties, normally expressed as:

$$\text{Log EC}10={\text{k}}_{1}\;\text{pH}+{\text{k}}_{2}\;\text{Log CEC}+{\text{k}}_{3}\;\text{Log OC}+\text{k}$$ (1)

where k_1_, k_2_ and k_3_ are constants for pH, cation exchange capacity (CEC) and organic carbon (OC) of soil in the Cu ecotoxicity predictive models, respectively. As an additional selection criterion, the soil properties affecting the bioavailability of Cu (i.e. pH, OC and CEC) fell into the range of the Cu ecotoxicity predictive models, i.e. under conditions for which the models have been demonstrated to accurately predict Cu ecotoxicity [17]. For different ecological endpoints, the EC10 retained was the most sensitive EC10 (the lowest value) identified for the test species. If one species had several EC10 values for the same endpoint available, these values were averaged (using a geometric mean) to derive the ‘species mean’ of EC10. All EC10 values were expressed related to EC10_add_, which did not include the background concentration. The final compiled soil Cu ecotoxicological database contained high-quality chronic ecotoxicity data for 21 different species belonging to different taxonomic groups, i.e. higher plants (19 different species covering several families) and two microbial processes. The database complied with the minimum quantity requirement of the SSD method–“at least 10 values (preferably more than 15) for different species” [27].

### Data handling and normalization

#### Leaching factor

Spiking a soil with a soluble metal salt affects the metal bioavailability and ecotoxicity in soil by increasing the ionic strength and decreasing soil pH [19,22]. Metal ecotoxicity in freshly spiked soils clearly differs from that in leached or aged soils [28], so a leaching procedure before ecotoxicity tests is recommended to mimic more realistic exposure conditions [29,30]. To determine the influence of soil leaching on Cu ecotoxicity, Li et al. [14,17,31] carried out a series of Cu ecotoxicity tests based on 17 Chinese soils with varying soil properties using barley root elongation, tomato and bok choy growth as the ecotoxicity endpoints. The tested soils were split into leached (leached with artificial rainwater before ecotoxicity test [19]) and unleached treatments, and the values of Cu EC50 (the concentrations that cause 50% inhibition effect) and EC10 for the three endpoints in the two treatments were obtained. The ratios of EC50 in leached soil to EC50 in unleached soils per endpoint per soil were calculated because EC50 values are more robust and have less experimental variation compared to EC10 [23]. The average value of the ratios for three endpoints in a soil was used as a leaching factor (LF) per soil in the present study. The ecotoxicity data from literature in which there was no leaching procedure before the ecotoxicity test were corrected by LF, i.e. multiplying the EC10 obtained from the ecotoxicity tests based on unleached soil by LF to get corrected EC10 values.

#### Aging factor

Ecotoxicity tests with plants, invertebrates and microbial response have demonstrated differences in Cu availability and ecotoxicity between freshly spiked and aged soils [19,32–34]. The ecotoxicity of added Cu decreased with increasing time between the addition of Cu to soils and the measurement of ecotoxicity [19]. The effect of time on metal bioavailability (aging effect) should be corrected to minimize the discrepancy caused by the difference between laboratory and field conditions. Aging effects of added Cu in soil were determined on a number of Cu-salt-amended soils that were incubated outdoors after leaching with simulated rainwater, and the isotopically exchangeable Cu concentrations (E values) were measured at regular intervals after amendment, with a maximum aging time of 2 years [35]. The research results indicated that incubation time and soil pH were the two most important factors for predicting the lability of Cu added to soils. The semi-mechanistic model for long-term aging of soluble Cu added to soils based on incubation time and soil pH was as follows[35]:

$$\text{E value}(\%)=100\;-\frac{89.8}{10^{\left(7.7-\text{pH}\right)}\;+1}\times {\text{t}}^{\frac{1}{\text{t}}}\;-\;4.92\times \text{ln}\left(\text{t}\right)$$ (2)

where E value (%) represents the percentage of E value of added Cu to total Cu added to soil, pH is soil pH measured in 0.01 M CaCl_2_ and t is aging time (d). Since most of the compiled data came from laboratory tests carried out two weeks after spiking of soils with soluble Cu salts, the value of the aging factor (AF) was set equal to the ratio of E value for 14 d (2 weeks) to that for 360 d (1 year). All compiled Cu EC10 values (from leached soil or corrected with LF) were multiplied by AF for correction.

#### Determination of representative scenarios of Chinese soil

To study the cross-extrapolation of Cu predictive models and then compare SSD curves distributions in different soils, the property information of the 17 soils sampled from the main agricultural areas in China were put into K-means cluster analysis to obtain representative scenarios of Chinese soils. Soil pH, CEC, clay content and OC content were chosen as the independent input variables for the K-means cluster analysis. These parameters were chosen because of their significant influence on the bioavailability/ecotoxicity of metals in soils [36]. In view of the soil property ranges in the monitoring data from the second national soil survey for 1982–1994 in China, the scope was set as 4.5–9.0 for pH, 5–30 cmol/kg for CEC and 1–4% for OC content, and so 240 artificial soil-specific conditions were obtained by combining pH, CEC and OC.

#### Selection of predictive models

The developed empirical predictive models were used to normalize Cu ecotoxicity data to modify the effects of soil properties in Cu-salt-amended soils. Since the ecotoxicity database contained EC10 values for species other than those for which specific predictive models have been developed, it is ideal to develop a separate species-specific predictive model for each species, but it is not considered realistic. The ability of predicting and the feasibility of normalizing ecotoxicity data for non-model organisms should be supported by quantitative evidence. We determined the ability of predictive models developed for tomato, barley and bok choy to predict Cu ecotoxicity for eight plant species for which no specific models had been developed. For the same scientific reason as choosing EC50 values to calculate LF mentioned above, the EC50 values and the predictive models of Cu EC50 for tomato, barley and bok choy were applied to validate the cross-species extrapolation of predictive models. This was under the assumption that the parameters, which were obtained for model organisms to describe the effect of soil properties, were constant across related species (within broad taxonomic groups). Thus, the constants k_1_, k_2_ and k_3_ were assumed to be the same among related species and the difference between related species was assumed to be their intrinsic sensitivity (k) [see Eq (1)]. Following these assumptions, we fitted the nominal intrinsic sensitivity (k) of each organism that resulted in the lowest sum of deviations between observed and predicted EC50 values [deviation = absolute value (observed EC50 –predicted EC50)] using available ecotoxicity data for eight plant species. The accuracy of the model predictions was evaluated by comparing the observed EC50 for each of the non-model organisms with the predicted EC50 using the model with the calculated k. This procedure was identical to that used in cross-extrapolation of Biotic Ligand Models of chronic nickel ecotoxicity [37].

The variation of Cu EC50 values for a particular organism originated partly from the difference in soil properties. Thus, the intra-species variability of EC50 values, which was expressed as coefficient of variation (CV), should decrease after normalization using the appropriate predictive model. Each EC50 value for the eight plant species was normalized individually to the soil with pH (7.0), CEC (15 cmol/kg) and OC (1.5%), which represents neutral soil properties in China [38]. Because the predictive models reduced intra-species variability this enabled comparison of intra-species variability between non-normalized EC50s and EC50s normalized to the particular soil properties.

#### Normalization of Cu EC10

All corrected Cu EC10s (i.e. EC10 values corrected with AF or with LF and AF) were normalized to soil-specific properties before being used as input data for the soil-specific SSD curve fitting. The normalization procedure itself takes into account the impact of soil properties on metal ecotoxicity and has been used in risk assessment [39,40]. The normalization of a single EC10_i, x_ obtained for a species i in a given test soil x to a ‘target soil’ with properties y was calculated as follows:

$$EC10_{i,y}=EC10_{i,x}+k_{1}(pH_{y}-pH_{x})+k_{2}\cdot Log(\frac{CEC_{y}}{CEC_{x}})+k_{3}\cdot Log(\frac{OC_{y}}{OC_{x}})$$ (3)

where k_1_, k_2_ and k_3_ are as defined in ‘Ecotoxicological Data Collection and Compilation’ (see Table 1). All EC10 values for Cu were normalized to the representative scenarios obtained by K-means cluster analysis mentioned above and the combined 240 artificial soil conditions. This normalization procedure yielded the input EC10 values (normalized EC10 per species) for the subsequent construction of Cu SSD curves per soil.

**Table 1:** Copper ecotoxicity predictive models used for ecotoxicological data normalization.

Species	Regression model (not aged)	R^2^	Reference
**Tomato, cucumber, green chilli, celery, spinach, Chinese cabbage, flowering cabbage, Chinese kale, eggplant, pechay and *Alternanthera philoxeroides***	LogEC10 = 0.635 + 0.092pH+ 0.873logCEC	0.56	[14,31]
**Bok choy**	LogEC10 = 1.554 + 0.706logOC	0.56	[14,31]
**Barley, wheat, rice, onion, mustard, radish and cabbage**	LogEC10 = 1.18 + 0.159pH+ 0.597logOC + 0.702logCEC	0.83	[17,31]
**Bioluminescent bacteria (Q67)**	LogEC10 = 0.411pH + 0.033CEC– 0.942	0.66	[15]
**Substrate-induced respiration**	LogEC10 = 0.565pH + 0.283OC– 2.247	0.58	*

* Obtained by multiple regression analysis of raw data from Ph.D. thesis of Li [41].

### SSD construction and calculation of HCx values

Several approaches to construct SSD curves have evolved over recent years. Differences between these approaches lie in the choice of underlying distribution such as the log-normal, log-logistic or Burr III [27]. The goodness-of-fit for different distributions had been compared and the result showed that Burr III fitted better than other distributions with smaller root mean square error (RMSE) [42]. Also the Burr III distribution has been reported to be a very flexible three-parameter distribution, which can provide good approximations to many commonly used distributions including the log-normal distribution [43].The three-parameter distribution Burr III was used to fit the normalized species-specific EC10 values to construct SSD curves, and then the hazardous concentration for x% of the species (HCx) was estimated in the present study. Due to uncertainty, the HCx at its 50% confidence level was used and denoted as HCx. According to the level of protection for different types of land, HCx was defined as follows: HC1 for national natural reserve, HC5 for agricultural and forestry land (including pasture), HC20 for urban residential and open areas and HC40 for commercial and industrial land. The selection of the percentile p is not a scientifically based decision but is a regulatory choice and determines the acceptable level of risk. The ecological investigation levels for Australian soil guidelines were also derived using a similar approach, in which HC1, HC20 and HC40 were chosen for pristine environments, urban residential and/or open space, and commercial and/or industrial areas, respectively [44].

### Calculation of ecological criteria for Cu and development of their predictive models

The ecological criteria for Cu, expressed as predicted no effect concentration (PNEC), was the scientific basis for the establishment of soil EQS to protect soil ecosystem. The approach of deriving PNEC from HCx has been adopted by different jurisdictions, including the Registration Evaluation Authorization and Restriction of Chemicals Regulation of the European Union, the National Environment Protection Council in Australia and the Environmental Protection Agency in the United States [43–45]. Normally an assessment factor (≥ 1) is utilized to lower PNEC, i.e. PNEC = HCx/assessment factor, when HCx values are derived from ecotoxicity data with few species or poor quality, or information on extrapolation from laboratory data to field conditions is limited. In the present study, HCx values were derived based on ecotoxicity data from a wide range of relevant native species and the soil bioavailability, leaching and aging were all taken into account in the derivation process to reduce differences between laboratory and field conditions. Thus, no additional assessment factor between the HCx and ecological criteria was used here (i.e. PNEC = HCx). All soil-specific HCx values for the 240 artificial soil conditions and their corresponding property information were chosen as input data for Excel Solver with the given condition that resulted in the lowest sum of deviations between derived and predicted HCx values [deviation = absolute value (derived HCx–predicted HCx)] to develop the predictive model for Cu ecological criteria.

## Results and Discussion

### Cross-species extrapolation of Cu ecotoxicity predictive models

**Fig 2:**
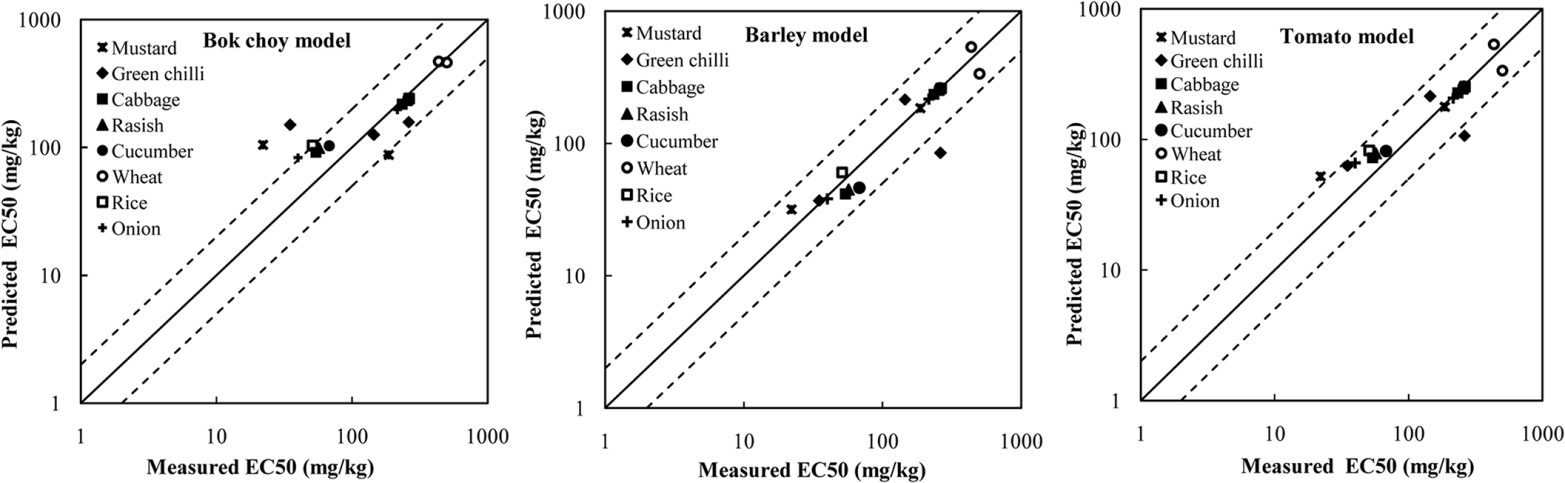
Relativity between the measured and predicted EC50 values of Cu.

**Fig 3:**
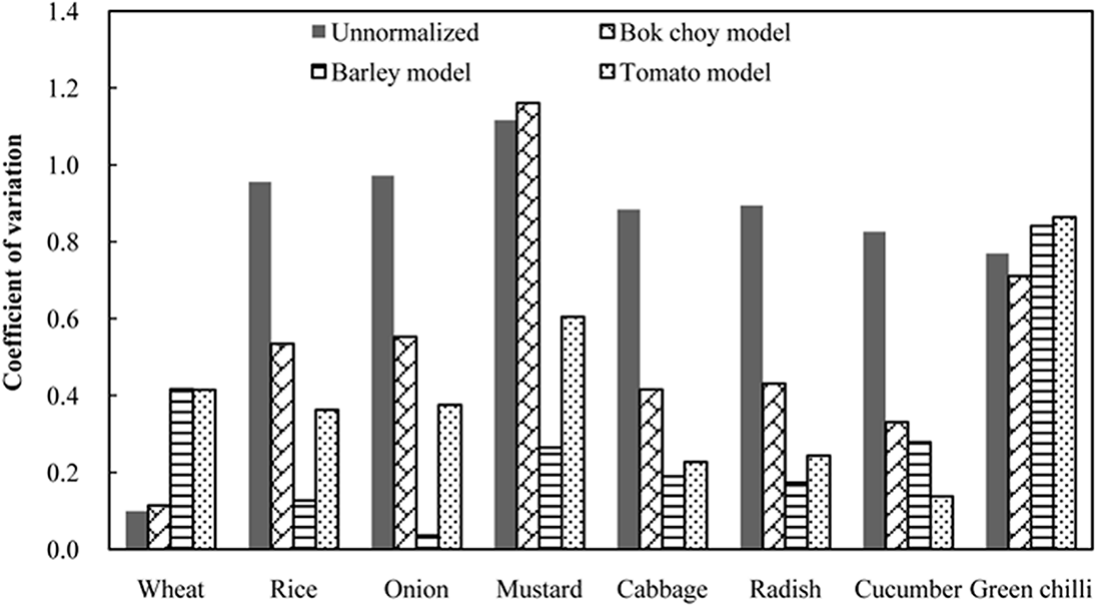
Intra-species variability of Cu EC50 values.

The predicted EC50s for rice, onion, radish, mustard and cabbage were accurate within a factor of 2.0 using the barley model (Fig 2). The bok choy model could predict the EC50s for wheat, cucumber and green chilli more accurately than the other two models (Fig 2). However, it is not rational to apply the bok choy model, a model that does not consider soil pH, to normalize ecotoxicity data for other plant species since soil pH significantly affects Cu ecotoxicity [17,18]. Although intra-species variability of EC50 for wheat increased after normalization using the barley model, the EC50 for wheat was still normalized with the barley model because they are similar monocotyledonous species. The EC50s for cucumber and green chilli were normalized using the tomato model. Most of the intra-species variabilities of the ecotoxicity data were substantially reduced for the non-model species following normalization (Fig 3). The exception was green chilli, for which the CV of normalized data was similar (only 9% higher) to that for non-normalized data. That may have been due to the low intra-species variability of non-normalized data for green chilli (i.e. 77%). The barley model reduced the intra-species variability of EC50s for rice, onion, mustard, cabbage and radish more than the other two models, with CV from 0.04 (onion) to 0.26 (mustard), which was consistent with the prediction accuracy of applying the barley model to these species. The tomato model reduced the intra-species variability of EC50 for cucumber by the most, with CV decreasing from 0.83 to 0.14 after normalization. The predictive models were chosen to normalize the ecotoxicity data for the eight plant species based on the prediction accuracy and the ability of reducing the intra-species variability. A trophic-level specific predictive model was applied to the other non-model species which had insufficient ecotoxicity data for model selection (e.g. a tomato model was used for celery). Cross-species extrapolation represents an improvement over no normalization, because it treats all ecotoxicity data within defined taxonomic categories consistently, which removes the influence of soil on bioavailability from the SSD [45]. The predictive models applied in the normalization procedure are listed in Table 1.

### Representative scenarios and SSD for Cu in Chinese soil

The K-means cluster analysis result of the 17 soil samples showed that Chinese soils can be classified in four types–i.e. acidic, neutral, alkaline calcareous and alkaline non-calcareous soils–with representative scenarios shown in Table 2.

**Table 2:** Four representative scenarios of Chinese soils.

Soil type	pH	CEC (cmol/kg)	OC (%)	Clay (%)
**Acidic**	5.0	10	1.0	55
**Neutral (including paddy)**	7.0	15	1.5	35
**Alkaline calcareous**	8.5	10	1.0	20
**Alkaline non-calcareous**	7.5	25	3.0	35

**Fig 4:**
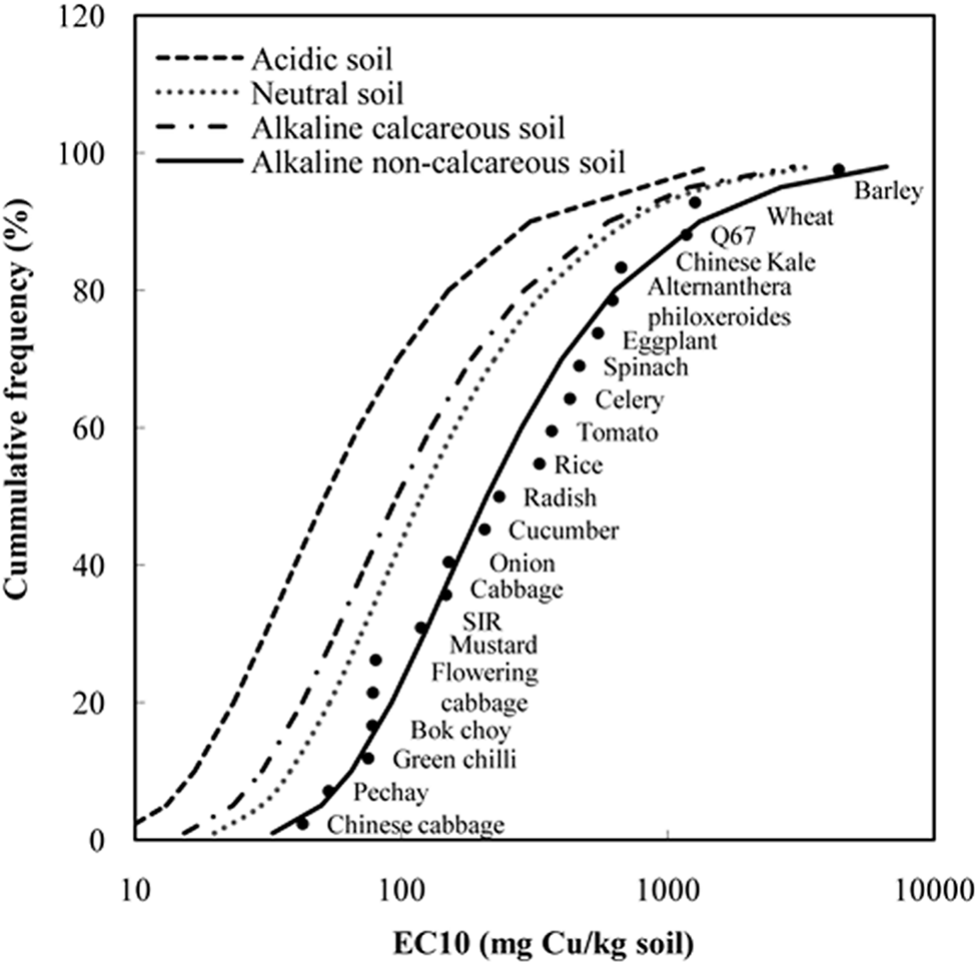
The Cu SSD curves fitted by Burr III functions for four representative scenarios of Chinese soils. The dots in the figure are Cu EC10 ecotoxicity data normalized to alkaline non-calcareous soil condition. SIR is substrate-induced respiration assay. Q67 is a toxicity test using bioluminescent bacteria *Vibrio qinghaiensis*.

The SSD curves for Cu in the four representative scenarios were constructed (Fig 4) by fitting normalized ecotoxicity data with Burr III. The figures visualized the order of sensitivity to Cu ecotoxicity among different species. Although some species exhibited slightly different sensitivities to added Cu in different soils, generally the order of species sensitivity was similar to that in alkaline non-calcareous soil (Fig 4) across the four different representative soil scenarios (Table 2). Chinese cabbage was the most sensitive species in the four scenarios, and vegetables had greater sensitivity than grain crops and Q67, as also mentioned by Li et al. [14]. It is rational to construct SSD curves based on ecotoxicity data on individual plant, micro-organisms and soil animals separately. But the available data on microbial species/processes and soil animals are few and do not comply with the minimum quantity of the SSD method. Actually, the SSD approach is a statistical extrapolation method and the HCx values derived from the SSD curves depend on ecotoxicity data of the number of species and their sensitivity to certain contaminant toxicity. SIR is a microbial process which is not sensitive to Cu ecotoxicity and has no significant effect on the value of HCx when we used ecotoxicity data of Q67 and SIR together with data on terrestrial species to construct the SSD curves. The HC5 values varied about fourfold among the different soils, i.e. between 13.1 mg/kg (acidic soils) and 51.9 mg/kg (alkaline non-calcareous soils).

### Major soil factors affecting ecological criteria for Cu and the predictive models

**Fig 5:**
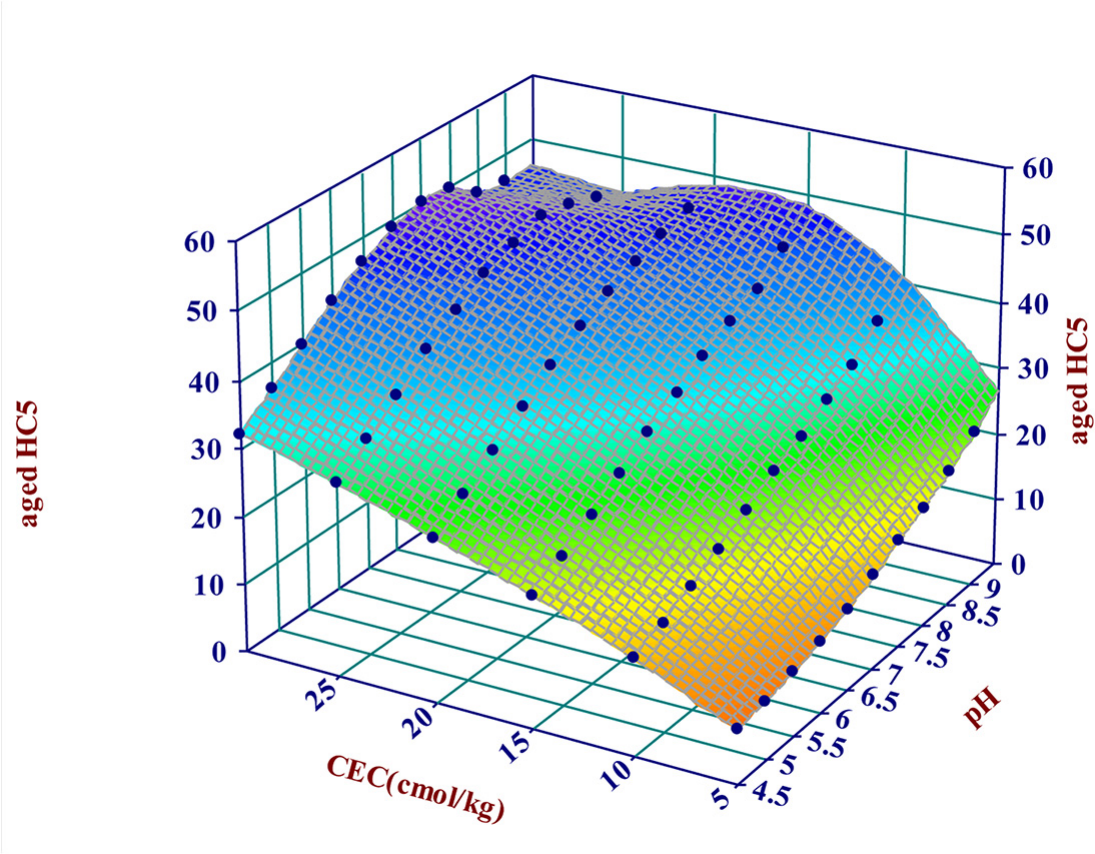
The impact of soil pH and CEC on Cu HC5 (OC was set at 1%).

The effects of pH and CEC on derived HC5 are shown in Fig 5. Partial correlative analyses based on the calculated HC5 values for all 240 artificial soil scenarios showed that CEC was the main variable affecting HC5 (r = 0.79) followed by soil pH (r = 0.53). In the scenarios with soil pH 7.0 and OC content of 1%, the Cu ecological criteria varied from 7.8 to 23.5 mg/kg at CEC of 5.0 and 30.0 cmol/kg, respectively. Similarly, with CEC and OC content constant, Cu ecological criteria were 14.0 and 43.9 mg/kg at pH 4.5 and 9.0, respectively.

Models for Cu ecological criteria were developed using Excel Solver based on the 240 properties-specific HCx values for artificial soils. The predictive models are shown in Table 3. The two-factor predictive models based on soil pH and CEC predicted Cu ecological criteria with determination coefficients (R^2^) of 0.82–0.91. The three-factor predictive models, taking into account the effect of OC on Cu ecological criteria, were more accurate than the two-factor models with R^2^ of 0.85–0.99. Soil pH and CEC explained > 80% of the variation in HCx, OC explained the variation in HCx lower than 10% with 3.2% for HC1, 6.7% for HC5, 8.7% for HC20 and 7.5% for HC40, respectively. The impact of soil properties on the magnitude of HCx reflects their effect on Cu ecotoxicity for soil organisms. This result differed from previous reports, which implied that the pH and OC were the main factors affecting Cu ecotoxicity in soil. Most Chinese soils are dominated by variable charge surfaces, are often depleted in organic matter and the values of CEC are affected by soil pH, OC, type and quantity of soil clay [46–48]. The significant effect of CEC on the magnitude of Cu HCx may be due to the other soil properties. The ecological criteria calculated with the two-factor model were lower than those derived based on ecotoxicity data in alkaline soils (pH ≥ 7.5) with high CEC. The three-factor models had prediction errors only in the soils with extreme physico-chemical conditions (i.e. pH ≥ 8.5 and CEC ≥ 25 cmol/kg). The CEC values in this study were measured with the method of neutral (pH 7) ammonium chloride buffered solution. Since all soils contain variable charge soil particles (pH dependent), when the pH of soil is altered to the pH value of the extracting solution during the CEC measurement, the resulting CEC values are different from actual field conditions. The magnitude of the change in CEC value depends on the difference in pH values between the soil and extracting solution. The characteristics of 17 Chinese agricultural soils showed that for soils with high pH the CEC values were normally < 20 cmol/kg and extreme physico-chemical condition were rare.

**Table 3:** Predictive models of Cu ecological criteria in soils for different land types.

Land type	Ecological criteria	Predictive model	
		Three-factor	Two-factor
**National natural reserve**	HC1	LogHC1 = 0.079pH + 0.176LogOC + 0.836LogCEC– 0.299 (R^2^ = 0.85)	LogHC1 = 0.076pH + 0.817LogCEC– 0.189 (R^2^ = 0.82)
**Agricultural and forestry land (including pasture)**	HC5	Log HC5 = 0.077pH + 0.231LogOC + 0.734LogCEC + 0.062 (R^2^ = 0.96)	LogHC5 = 0.076pH + 0.733LogCEC + 0.172 (R^2^ = 0.89)
**Urban residential and open areas**	HC20	LogHC20 = 0.083pH + 0.259LogOC + 0.667LogCEC + 0.407 (R^2^ = 0.99)	LogHC20 = 0.083pH + 0.667LogCEC + 0.499 (R^2^ = 0.91)
**Commercial and industrial areas**	HC40	LogHC40 = 0.094pH + 0.249LogOC + 0.672LogCEC + 0.583 (R^2^ = 0.99)	LogHC40 = 0.095pH + 0.677LogCEC + 0.659 (R^2^ = 0.91)

### Validation and application of Cu ecological criteria

**Fig 6:**
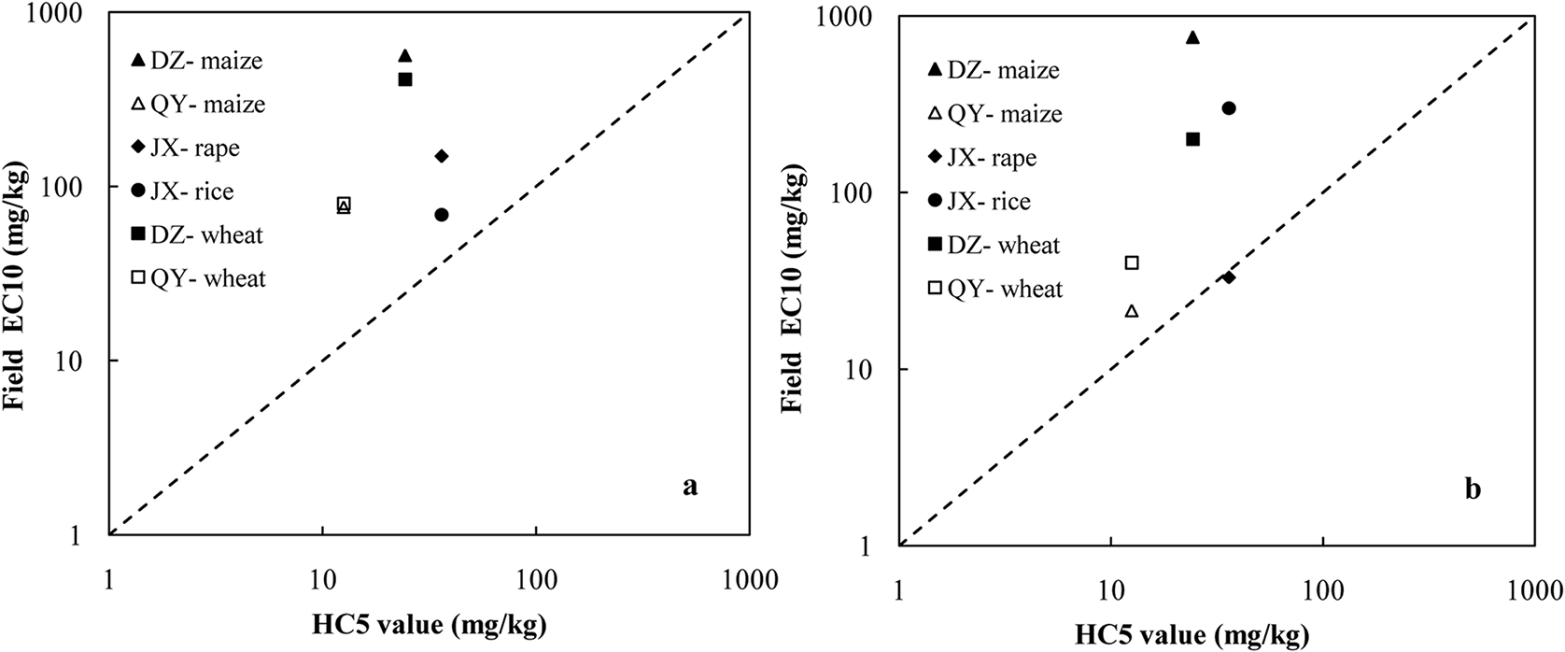
Comparison of HC5 derived using ecotoxicity data from different field crops at three field sites. The ecotoxicity data were obtained from field experiments conducted in three field sites from Qiyang in Hunan (QY) as acidic soil, Dezhou in Shandong (DZ) as alkaline soil, and Jiaxing in Zhejiang (JX) as neutral soil in 2007 (a) and 2008 (b).

Three field crop experiments were carried out to investigate dose–response relationships of added Cu to soils of contrasting soil properties to identify the reliability of laboratory bioassays for field crops. The three field experimental sites were from Qiyang in Hunan (QY) on an acidic soil, Dezhou in Shandong (DZ) on an alkaline soil, and Jiaxing in Zhejiang (JX) on a neutral soil [49–51]. A series of two-year field experiments were conducted to study the phytotoxicity of Cu added to soils with a maize–wheat rotation in Qiyang and Dezhou and with a rice–rape rotation in Jiaxing. The grain yield was used as the ecotoxicity endpoint, and the Cu EC10 values for these field crops were obtained by fitting dose–response data with a logistic model [52]. Comparing the derived Cu ecological criteria for agricultural soil with Cu EC10 values of these field crops demonstrated that ecological criteria derived from the bioavailability-normalized laboratory ecotoxicity data were protective (i.e. conservative) compared to the effects under field conditions using highly soluble Cu salts as the source of soil contamination, even for sensitive plant species (i.e. rape) (Fig 6). Environmental metal contamination seldom occurs with fully soluble metal sources. Luo et al. [4] showed that livestock manures are responsible for 69% of the total Cu input to agricultural soils in China. The ecotoxicity of Cu applied to soils in the form of soluble salts is much greater than that from animal manures or other common sources of Cu contamination (e.g. biosolids, composts, slag and atmospheric deposition of Cu-particulates) because of the low availability of Cu in these solids [32,33]. Therefore, the ecological criteria derived from laboratory bioassays using addition of Cu salts as contamination sources are conservative for field soil ecosystems.

The Cu in surface soils is derived from both parent material and anthropogenic activities and so it is often difficult to quantify the natural background Cu concentration in soils. It is more pragmatic to use a critical value which is inclusive of background concentrations in soil EQS establishment and risk assessment. A continuous PNEC based on total concentration (PNEC_total_) could be calculated as the sum of ecological criteria (PNEC derived here) and the soil-specific background concentration (Cb). It is very convenient to apply PNEC_total_ in a regional environmental assessment of Cu for soils because the Cu ecological criteria can be calculated by the predictive models mentioned above based on soil property parameters, and the background concentration can be inferred from the Cu concentration in a clean reference site with a comparable soil type to the site being assessed. The PNEC_total_ derived by the approach provided in the present study is more scientific than the current Chinese soil EQS for Cu, which used invariant total Cu concentrations in two pH ranges (50 mg/kg for pH < 6.5 and 100 mg/kg for pH ≥ 6.5) as limit values without taking into account the difference of background concentrations in soils, the quantitative relationship between toxicity and soil properties effects and differences in sensitivity among species. In the practice of soil pollution monitoring and controlling, implementation of PNEC_total_ will provide a realistic and accurate risk level for the soil being assessed.

## Conclusions

For incorporation into ecotoxicity predictive models, the Cu ecological criteria (which were set equal to HCx) were derived with ecotoxicity data based on SSD in the added risk approach. Soil properties have a significant effect on Cu bioavailability and hence CEC, pH and OC could explain > 80% of the variation in HCx. The predictive models derived in this study could be used to accurately calculate continuous Cu ecological criteria based on soil properties and allow Cu pollution risk assessment to be site or soil specific. This is a significant improvement on the traditional approach, which applies a single or multi-stage limit value, and so minimizes both over- and under-protection of terrestrial ecosystems. The strength of the ecological criteria derivation approach used in the present study is that it is risk-based and enables protection of a selected percentage of species. The ecological criteria derived here accommodate ecotoxicity data from a wide range of local species, land uses and purposes, and also incorporate bioavailability considerations. However, the Cu ecotoxicity data used in this study came from laboratory bioassays conducted using soil spiked with Cu salts. The limitation of the approach adopted here is that it does not account for bioavailability differences arising from different sources, forms or speciation of contaminants. Although leaching and aging factors were applied to reduce the difference between laboratory and field conditions, the ecological criteria derived in this study are conservative, since the ecotoxicity of Cu applied to soils as soluble salts is much greater than that from other common sources of Cu contamination. Additional research on the relationship between Cu ecotoxicity in soil spiked with Cu salts and other contamination resources should be carried out to make ecological criteria more realistic. As Cu is one of the naturally occurring elements with widely varying background concentrations, a reliable methodology to determine the ambient background Cu concentration in soils will contribute to a more science-based Cu pollution risk assessment.

## Supporting Information

S1 File Normalization and ecotoxicity dataset of copper in soils. (DOC)
