# Correction: Derivation of Soil Ecological Criteria for Copper in Chinese Soils

**DOI:** 10.1371/journal.pone.0140306

**Published:** 2015-10-06

**Authors:** Xiaoqing Wang, Dongpu Wei, Yibing Ma, Mike J. McLaughlin

Fig 1 is incorrect. The order of “Fit SSD curves and calculate HCx” and “Normalize corrected data with Cu ecotoxicity predictive models” should be switched. The authors have provided a corrected version here.

**Fig 1:**
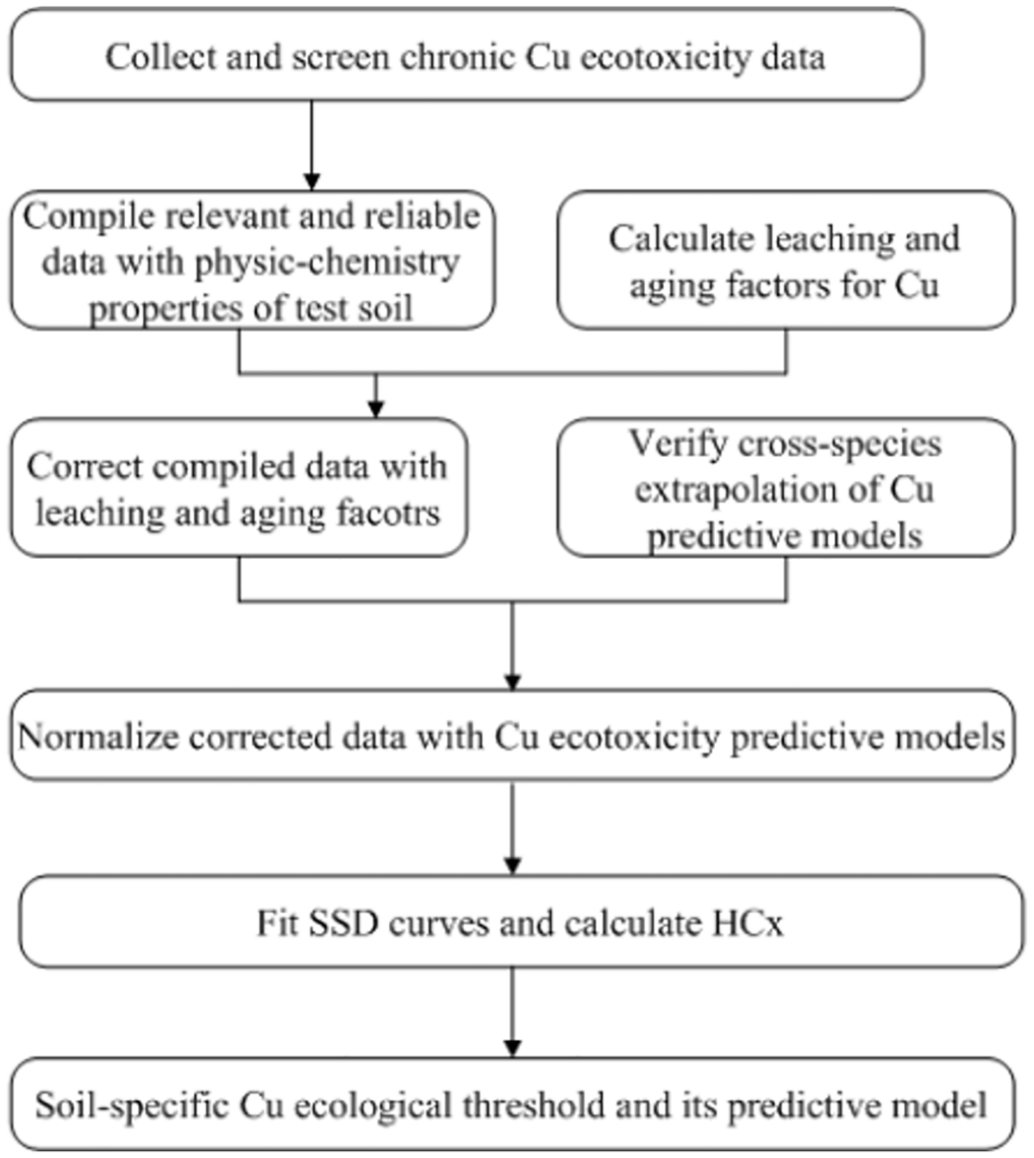
Schematic framework used for derivation of soil Cu ecological criteria.
