# Supplementary material for: Derivation of Soil Ecological Criteria for Copper in Chinese Soils
Source: PLoS One. 2015 Jul 24;10(7):e0133941. doi: 10.1371/journal.pone.0133941 (PMC4514794; doi:10.1371/journal.pone.0133941)
Supplement: S1 File — (DOC) [file pone.0133941.s001.doc]

**Normalization and ecotoxicity dataset of copper in soils**

The information on literature data transformation by applying aging/leaching factors and toxicity normalization models has been given as below:

1. The leaching factors were calculated with raw data of Li Bo’s Ph D thesis [1], and described in the part of “Materials and methods” at lines 138-149.
2. The toxicity normalization models were refereed from the references noted in Table 1 and listed in the part of “References” of manuscript.
3. The original literatures for Cu ecotoxicity data selection were provided in References for data sources for Chinese ecotoxicity data in the supporting information..

The final selected data used for deriving soil criteria were listed in Tables A and B.

Table A. Toxicity data of higher plants from leached soils (unless otherwise indicated)

| Site | pH | OC (%) | CEC  (cmol/kg) | Clay  (%) | Organism | Endpoint | EC10 (mg/kg) |
| --- | --- | --- | --- | --- | --- | --- | --- |
| Chengdu [2] | 7.2 | 1.13 | - | - | Wheat | Shoot growth | 251.7 (unleached) |
| Fuzhou [3] | 4.87 | 1.56 | 6.9 | - | Chinese cabbage | Shoot growth | 5.42 (unleached) |
| Hangzhou [4] | 6.31 | 2.61 | 12.47 | 20.5 | Rice | Root length | 125 (unleached) |
| Hunan [5] | 4.9 | 1.48 | 5.9 | - | Tomato | Root length | 8 (unleached) |
| Jiaxing [5] | 6.0 | 4.56 | 16.1 | - | Tomato | Root length | 77 (unleached) |
| Beijing [5] | 8.1 | 1.96 | 23.8 | - | Tomato | Root length | 110 (unleached) |
| Dongguan [6] | 4.68 | 1.99 | 5.42 | - | Flowering cabbage | Shoot growth | 7.7 (unleached) |
| Hunan [7] | 4.94 | 1.49 | 5.9 | - | Bok choy | Root length | 8 (unleached) |
| Hunan [7] | 4.94 | 1.49 | 5.9 | - | Cabbage | Root length | 13 (unleached) |
| Hunan [7] | 4.94 | 1.49 | 5.9 | - | Cucumber | Root length | 24 (unleached) |
| Hunan [7] | 4.94 | 1.49 | 5.9 | - | Radish | Root length | 25 (unleached) |
| Hunan [7] | 4.94 | 1.49 | 5.9 | - | Tomato | Root length | 9 (unleached) |
| Hunan [7] | 4.94 | 1.49 | 5.9 | - | Onion | Root length | 10 (unleached) |
| Jiaxing [8] | 5.97 | 4.56 | 16.1 | - | Bok choy | Root length | 8 (unleached) |
| Jiaxing [8] | 5.97 | 4.56 | 16.1 | - | Cabbage | Root length | 13 (unleached) |
| Jiaxing [8] | 5.97 | 4.56 | 16.1 | - | Cucumber | Root length | 24 (unleached) |
| Jiaxing [8] | 5.97 | 4.56 | 16.1 | - | Radish | Root length | 25 (unleached) |
| Jiaxing [8] | 5.97 | 4.56 | 16.1 | - | Tomato | Root length | 9 (unleached) |
| Jiaxing [8] | 5.97 | 4.56 | 16.1 | - | Onion | Root length | 10 (unleached) |
| Shijiazhuang [9] | 8.36 | 1.16 | 7.43 | - | Wheat | Grain yield | 186 (unleached) |
| Shanxi [10] | 8.51 | 0.53 |  | - | Pechay | Shoot growth | 23.3 (unleached) |
| Fuzhou [11] | 6.57 | 0.93 | 9.87 | - | Green Chili | Grain yield | 33.8 (unleached) |
| Wuhu [12] | 7.31 | 1.36 | - | - | [Alternanthera](app:ds:alternanthera) [philoxeroides](app:ds:philoxeroides) | Plant length | 250 (unleached) |
| Wuhan [13] | 7.1 | 1.84 | 15.8 | - | Chinese kale | Shoot growth | 231.6 (unleached) |
| Shenyang [14] | 6.9 | 0.66 | - | - | Eggplant | Grain yield | 213.5 (unleached) |
| Hainan | 4.93 | 1.51 | 8.75 | 66 | Barley | Root elongation | 54 |
| Hunan | 5.31 | 0.87 | 7.47 | 46 | Barley | Root elongation | 38 |
| Hailun | 6.56 | 3.03 | 33.59 | 40 | Barley | Root elongation | 446 |
| Jiaxing | 6.70 | 1.42 | 19.33 | 41 | Barley | Root elongation | 121 |
| Hangzhou | 6.80 | 2.46 | 12.83 | 39 | Barley | Root elongation | 145 |
| Chongqing | 7.12 | 0.99 | 22.30 | 27 | Barley | Root elongation | 187 |
| Guangzhou | 7.27 | 1.47 | 8.30 | 25 | Barley | Root elongation | 177 |
| Beijing | 7.48 | 4.28 | 22.65 | 20 | Barley | Root elongation | 715 |
| Neimeng | 7.66 | 2.66 | 22.74 | 37 | Barley | Root elongation | 647 |
| Jilin | 7.82 | 2.17 | 28.76 | 45 | Barley | Root elongation | 287 |
| Shijiazhuang | 8.19 | 1.00 | 11.71 | 21 | Barley | Root elongation | 106 |
| Xinjiang | 8.72 | 0.87 | 10.31 | 25 | Barley | Root elongation | 209 |
| Shanxi | 8.83 | 0.62 | 8.46 | 27 | Barley | Root elongation | 154 |
| Langfang | 8.84 | 0.60 | 6.36 | 10 | Barley | Root elongation | 112 |
| Zhengzhou | 8.86 | 1.57 | 8.50 | 16 | Barley | Root elongation | 96 |
| Gansu | 8.86 | 1.02 | 8.08 | 20 | Barley | Root elongation | 215 |
| Shandong | 8.90 | 0.69 | 8.33 | 18 | Barley | Root elongation | 187 |
| Zhangzhou [15] | 4.70 | 1.23 | 9.0 | - | Barley | Root elongation | 34.8 |
| Haikou [15] | 5.60 | 0.656 | 12.35 | - | Barley | Root elongation | 112.0 |
| Shanghai [15] | 7.01 | 0.887 | 16.91 | - | Barley | Root elongation | 101.2 |
| Tongren [15] | 6.65 | 1.032 | 16.19 | - | Barley | Root elongation | 150.5 |
| Yantai [15] | 7.22 | 1.62 | 19.46 | - | Barley | Root elongation | 80.5 |
| Wuwei [15] | 7.08 | 1.819 | 11.72 | - | Barley | Root elongation | 106.1 |
| Luoyang [15] | 7.44 | 0.597 | 12.01 | - | Barley | Root elongation | 193.0 |
| Naqu [15] | 7.35 | 1.25 | 8.43 | - | Barley | Root elongation | 96.8 |
| Hainan | 4.93 | 1.51 | 8.75 | 66 | Tomato | Shoot biomass | 79 |
| Hunan | 5.31 | 0.87 | 7.47 | 46 | Tomato | Shoot biomass | 76 |
| Hailun | 6.56 | 3.03 | 33.59 | 40 | Tomato | Shoot biomass | 651 |
| Jiaxing | 6.70 | 1.42 | 19.33 | 41 | Tomato | Shoot biomass | 208 |
| Hangzhou | 6.80 | 2.46 | 12.83 | 39 | Tomato | Shoot biomass | 164 |
| Chongqing | 7.12 | 0.99 | 22.30 | 27 | Tomato | Shoot biomass | 176 |
| Beijing | 7.48 | 4.28 | 22.65 | 20 | Tomato | Shoot biomass | 524 |
| Neimeng | 7.66 | 2.66 | 22.74 | 37 | Tomato | Shoot biomass | 610 |
| Jilin | 7.82 | 2.17 | 28.76 | 45 | Tomato | Shoot biomass | 173 |
| Shijiazhuang | 8.19 | 1.00 | 11.71 | 21 | Tomato | Shoot biomass | 137 |
| Xinjiang | 8.72 | 0.87 | 10.31 | 25 | Tomato | Shoot biomass | 298 |
| Shanxi | 8.83 | 0.62 | 8.46 | 27 | Tomato | Shoot biomass | 317 |
| Langfang | 8.84 | 0.60 | 6.36 | 10 | Tomato | Shoot biomass | 146 |
| Zhengzhou | 8.86 | 1.57 | 8.50 | 16 | Tomato | Shoot biomass | 164 |
| Gansu | 8.86 | 1.02 | 8.08 | 20 | Tomato | Shoot biomass | 187 |
| Shandong | 8.90 | 0.69 | 8.33 | 18 | Tomato | Shoot biomass | 124 |
| Hainan | 4.93 | 1.51 | 8.75 | 66 | Bok choy | Shoot biomass | 36 |
| Hunan | 5.31 | 0.87 | 7.47 | 46 | Bok choy | Shoot biomass | 23 |
| Hailun | 6.56 | 3.03 | 33.59 | 40 | Bok choy | Shoot biomass | 54 |
| Jiaxing | 6.70 | 1.42 | 19.33 | 41 | Bok choy | Shoot biomass | 46 |
| Hangzhou | 6.80 | 2.46 | 12.83 | 39 | Bok choy | Shoot biomass | 103 |
| Chongqing | 7.12 | 0.99 | 22.30 | 27 | Bok choy | Shoot biomass | 34 |
| Guangzhou | 7.27 | 1.47 | 8.30 | 25 | Bok choy | Shoot biomass | 57 |
| Beijing | 7.48 | 4.28 | 22.65 | 20 | Bok choy | Shoot biomass | 140 |
| Neimeng | 7.66 | 2.66 | 22.74 | 37 | Bok choy | Shoot biomass | 46 |
| Jilin | 7.82 | 2.17 | 28.76 | 45 | Bok choy | Shoot biomass | 90 |
| Shijiazhuang | 8.19 | 1.00 | 11.71 | 21 | Bok choy | Shoot biomass | 17 |
| Xinjiang | 8.72 | 0.87 | 10.31 | 25 | Bok choy | Shoot biomass | 27 |
| Shanxi | 8.83 | 0.62 | 8.46 | 27 | Bok choy | Shoot biomass | 41 |
| Langfang | 8.84 | 0.60 | 6.36 | 10 | Bok choy | Shoot biomass | 30 |
| Zhengzhou | 8.86 | 1.57 | 8.50 | 16 | Bok choy | Shoot biomass | 64 |
| Gansu | 8.86 | 1.02 | 8.08 | 20 | Bok choy | Shoot biomass | 29 |
| Shandong | 8.90 | 0.69 | 8.33 | 18 | Bok choy | Shoot biomass | 41 |

Note: the numbers 2-15 following the names of sites were the reference number for data source, otherwise all other toxicity data were from Li Bo’s Ph D thesis [1].

Table B. Toxicity data of microbial processes and Q67 from leached soils

| Site | pH | OC (%) | Clay (%) | CEC (cmol/kg) | Organism | Endpoint | EC10 (mg/kg) |
| --- | --- | --- | --- | --- | --- | --- | --- |
| Hainan | 4.93 | 1.5 | 8.75 | 66 | Q67^*^ | Bioluminescence | 50 |
| Hunan | 5.31 | 0.9 | 7.47 | 46 | Q67 | Bioluminescence | 38 |
| Hailun | 6.56 | 3 | 33.6 | 40 | Q67 | Bioluminescence | 851 |
| Jiaxing | 6.7 | 1.4 | 19.3 | 41 | Q67 | Bioluminescence | 651 |
| Hangzhou | 6.8 | 2.5 | 12.83 | 39 | Q67 | Bioluminescence | 167 |
| Chongqing | 7.12 | 1 | 22.3 | 27 | Q67 | Bioluminescence | 250 |
| Guangzhou | 7.27 | 1.5 | 8.3 | 25 | Q67 | Bioluminescence | 233 |
| Beijing | 7.48 | 4.3 | 22.6 | 20 | Q67 | Bioluminescence | 600 |
| Neimeng | 7.66 | 2.7 | 22.7 | 37 | Q67 | Bioluminescence | 197 |
| Jilin | 7.82 | 2.2 | 28.7 | 45 | Q67 | Bioluminescence | 1415 |
| Shijiazhuang | 8.19 | 1 | 11.7 | 21 | Q67 | Bioluminescence | 276 |
| Xinjiang | 8.72 | 0.9 | 10.3 | 25 | Q67 | Bioluminescence | 381 |
| Shanxi | 8.83 | 0.6 | 8.46 | 27 | Q67 | Bioluminescence | 1197 |
| Langfang | 8.84 | 0.6 | 6.36 | 10 | Q67 | Bioluminescence | 169 |
| Zhengzhou | 8.86 | 1.6 | 8.5 | 16 | Q67 | Bioluminescence | 1562 |
| Gansu | 8.86 | 1 | 8.08 | 20 | Q67 | Bioluminescence | 315 |
| Shandong | 8.9 | 0.7 | 8.33 | 18 | Q67 | Bioluminescence | 744 |
| Hainan | 4.93 | 1.5 | 8.75 | 66 |  | SIR^**^ | 160 |
| Hunan | 5.31 | 0.9 | 7.47 | 46 |  | SIR | 57.1 |
| Hailun | 6.56 | 3 | 33.6 | 40 |  | SIR | 214 |
| Jiaxing | 6.7 | 1.4 | 19.3 | 41 |  | SIR | 175 |
| Hangzhou | 6.8 | 2.5 | 12.8 | 39 |  | SIR | 681 |
| Chongqing | 7.12 | 1 | 22.3 | 27 |  | SIR | 104 |
| Guangzhou | 7.27 | 1.5 | 8.3 | 25 |  | SIR | 991 |
| Beijing | 7.48 | 4.3 | 22.6 | 20 |  | SIR | 65.8 |
| Neimeng | 7.66 | 2.7 | 22.7 | 37 |  | SIR | 262 |
| Jilin | 7.82 | 2.2 | 28.7 | 45 |  | SIR | 325 |
| Shijiazhuang | 8.19 | 1 | 11.7 | 21 |  | SIR | 13.0 |
| Xinjiang | 8.72 | 0.9 | 10.3 | 25 |  | SIR | 16.6 |
| Shanxi | 8.83 | 0.6 | 8.46 | 27 |  | SIR | 19.5 |
| Langfang | 8.84 | 0.6 | 6.36 | 10 |  | SIR | 18.9 |
| Zhengzhou | 8.86 | 1.6 | 8.5 | 16 |  | SIR | 10.7 |
| Gansu | 8.86 | 1 | 8.08 | 20 |  | SIR | 33.8 |
| Shandong | 8.9 | 0.7 | 8.33 | 18 |  | SIR | 20.5 |

Note: * The toxicity data were from Wei Dongpu’s Ph D thesis [16].

** The toxicity data were from Li Xiaofang’s Ph D thesis [17].

References for data sources for Chinese ecotoxicity data.

1. Li B. The Phytotoxicity of added copper and nickel to soils and predictive models. Ph D thesis, Chinese Academy of Agricultural Sciences. 2010. Available: http://epub.cnki.net. (in Chinese)
2. Zhang YL. Seed germination and seedling growth of *Triticum arstivum* under the stress of Cu and Pb. Thesis of master, Sichuan Normal University. 2008. Available: http://epub.cnki.net. (in Chinese)
3. Ye YS. Study on the toxicity of Cu to vegetables and the toxic threshold of soil Cu. Thesis of master, Fujian Agriculture and Forestry University 2009. Available: http://epub.cnki.net. (in Chinese)
4. Sun Q. Microbial ecological and crop effects and diagnostic index of copper pollution in grain-vegetable rotation ecosystems. Ph D thesis, Zhejiang University. 2008. Available: http://epub.cnki.net. (in Chinese)
5. Na ML, Xu MG, Zhang JX, Gong CY, Duan CL. Inhibit ion effect s of Cu, Zn and Pb on root elongation of tomato in three typical contaminated soils of China. Asian Journal of Ecotoxicology. 2008, 3(1): 81-86. (in Chinese)
6. Wang WH, Wu G, Liao ZW, Li JP. Effects of applying Cu, Zn to latored soil on the growth of *Brassica parachinensis*. J. South China Agr. Univ., 1996, 18(2): 66-71. (in Chinese)
7. Xu MG, Na ML, Zhang JX, Zhang WJX, Liu JL. Inhibition effects of Cu, Zn and Pb on vegetable root elongation in contaminated red soil. China Environmental Science. 2008, 28(2): 153-157. (in Chinese)
8. Zhang JX, Na ML, Xu MG. Inhibition and Toxicity of Cu, Zn, Pb on Root Elongation of Vegetable in Contaminated Soil. Journal of Agro-Environment Science. 2007, 26(3): 945- 949. (in Chinese)
9. Li HY, Chen S, Wang H. Study on fates of Cu and Zn in soil-plants system as well as their critical contents. Rural Eco-Environment. 1994, 10(22): 22-24. (in Chinese)
10. Yuan X. Effect of copper on small brassinca Chinensis growth and protective enzyme activity. Thesis of master, Northwest Agriculture and Forest University. 2008. Available: http://epub.cnki.net. (in Chinese)
11. Liu JC, Li YH, Jin H. Effects of copper contamination on yields, accumulated copper distribution and membrane protective enzyme activities of pepper. Fujian Journal of Agricultural Sciences. 2003, 18(4): 254-257. (in Chinese)
12. Huang YJ, Yang JH, Yang HF, Zhang J, Zhou SB. Effects of copper pollution on growth of [*alternanthera*](app:ds:alternanthera) [*philoxeroides*](app:ds:philoxeroides) and activity of soil enzymes. Acta Pedologica Sinica. 2009, 46(3): 494-500. (in Chinese)
13. Dai LP, Ke WS, Chen JJ, N R. Eco-toxicological response of *Brassica campestris L. var. purpurea Baileysh* to copper. Journal of Hubei University (Natural Science Edition). 2004, 26(2): 160-163. (in Chinese)
14. Yi YL, Liu SS, Zhang DG, Chen XL, L Y, Zhao MS. The effects of copper on eggplant yield and copper accumulation in brown soil. Northern Horticulture. 2010(5): 47-49. (in Chinese)
15. Li D, Yuan T, Guo GY, Wang WH. The copper bioavailability and their affecting factors in different soils. Environmental Science & Technology. 2007, 30(8): 6-9. (in Chinese)
16. Wei DP. The Application of Bioluminescent Bacteria Bioassay on Determination the Toxicity of Copper and Nickel in Chinese Soils. PhD thesis, Chinese Academy of Agricultural Sciences. 2010. Available: http://epub.cnki.net. (in Chinese)
17. Li XF. Copper and nickel toxicity in Chinese soils: their toxicity thresholds based on nitrification assay and ecological impacts on soil ecosystem. PhD thesis, Chinese Academy of Sciences. 2010. Available: http://epub.cnki.net. (in Chinese)
